# Dairy goat production in sub-Saharan Africa: current status, constraints and prospects for research and development

**DOI:** 10.5713/ajas.19.0377

**Published:** 2019-07-01

**Authors:** Alexander K. Kahi, Chrilukovian B. Wasike

**Affiliations:** 1 Animal Breeding and Genomics Group, Department of Animal Sciences, Egerton University, P.O. Box 536, 20115 Egerton, Kenya; 2 Livestock Efficiency Enhancement Group (LEEG), Department of Animal Science, Maseno University, P.O. Private Bag, 40105 Maseno, Kenya

**Keywords:** Dairy Goats, Sub-Saharan Africa, Milk Production, Research and Development, Low Input Systems

## Abstract

This paper presents a review of dairy goat production in sub-Saharan Africa (SSA) from 2010–2017, its current state, constraints and prospects for research and development. Since the introduction of dairy goats in SSA in pre-colonial times, their populations have continued to increase due to declining land size as a result of land fragmentation and increasing demand for goat milk. The current goat population in SSA is 372,716,040 head of which only 15.98% used for milk production. Populations in the Eastern and Western regions of SSA have shown an increasing trend from 2010 to 2017. The Southern Africa goat population is on the decline at an annual rate of about 1.77% whereas Central Africa has had a constant goat population within the same period. Eastern Africa reported the highest increase in the population of goats used for milk production. Milk production was highest in Eastern Africa and lowest in Southern Africa. However, dairy goat productivity remained constant in the Eastern region throughout the review period. Dairy goats are mainly raised under smallholder mixed crop-livestock systems. To enhance the development of the dairy goat, concerted efforts should be made to alleviate the constraints that stifle its growth. These constraints can be categorized into nutrition and feeding, breeding and reproduction, diseases, parasites, climate change, and underdeveloped dairy goat products market. Effective management of dairy goats requires a holistic approach and there is the need to expand the markets by further sensitization on the nutritional and medicinal advantages of dairy goat products. In order to achieve rapid development in the dairy goat sub sector, research and development initiatives should be directed towards alleviating the hurdles in nutrition and feeding, breeding, animal health and resilience as well as dairy goat markets.

## INTRODUCTION

Goats play important roles in the livelihoods of pastoralists in sub-Saharan Africa (SSA) [1–3]. These roles include provision of meat and milk as mainstream protein sources, blood used for food in some pastoralist communities, skins for the leather industry, economic safety net and acting as a bridge into production of large livestock [4,5]. The role of goats in provision of these services remains unparalleled especially in marginal areas owing to their high level of adaptability to harsh production conditions and continuous climatic changes [5] as well as the progressive increase in human population that is leading to decline in arable land. These roles are underestimated in many instances. Due to their adaptability, these animals can be found virtually in all agro-ecological zones in SSA.

Traditionally, indigenous goats are raised in SSA for meat although quite a number of them are also milked [3,5,6]. These goats have remained present in the arid and semi-arid areas where they continue to play the aforementioned roles. However, in highlands and humid lowlands, indigenous goats are primarily raised for meat while exotic dairy goat breeds are raised for milk [7,8].

The introduction of dairy goats in SSA dates back to pre-colonial times [7]. The breeds introduced include Saanen, Toggenberg, Alpine and Anglo-Nubian. Currently, the dairy goats stock consists of these breeds and their crossbred genotypes. Since their introduction, the number of dairy goats has continued to increase due to declining land sizes as a result of fragmentation to the extent that large ruminants cannot be raised. Also, there has been an increasing demand for goat milk resulting from increased sensitization of producers on the benefits of dairy goats by governments in collaboration with Non-Governmental Organisations such as FARM-Africa, Heifer International and World Vision, and bilateral agencies such as GIZ (*Deutsche Gesellschaft für Internationale Zusammenarbeit*) and United States Agency for International Development [9]. The efforts by these organizations have led to increased presence of dairy goats in the region.

Dairy goat production in SSA occurs under various production circumstances with different production opportunities and constraints. This results in variability in output levels and profitability that influence producer preference. Therefore, improvement of dairy goat production comes as a result of proper understanding of the challenges and opportunities available in the various production systems. This paper identifies the constraints and explores the prospects and possible intervention measures for the improvement of dairy goat production in SSA.

## STATUS OF GOAT PRODUCTION

### Milk goat populations and productivity

According to the FAOSTAT [10], goat population in the SSA stands at 372,716,040 head. Of these, only 59,567,125 accounting for 15.98% of the total are goats used for milk production. Table 1 shows the population of goats and their proportionate distribution in SSA. Goat populations in SSA regions vary significantly. Western Africa has the largest number of goats while Southern Africa has the lowest goat population in the region. A similar observation is made for the dairy goat population, with the number used for milk production being highest (29,318,272) in Western Africa and lowest (110,693) in Southern Africa. The high numbers in Western Africa could be attributed to majority of goats used for milk being indigenous whose productivity is relatively low to Southern Africa

**Figure 1:**
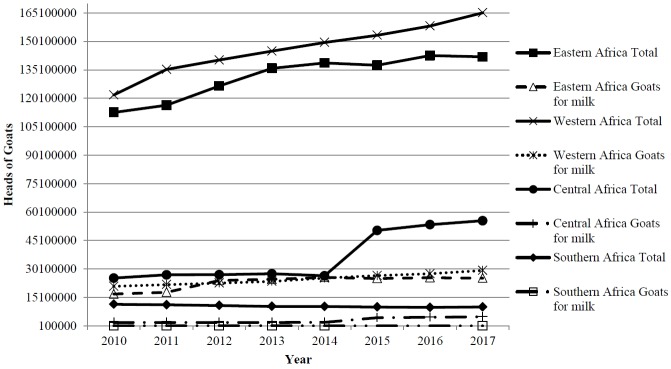
Goat population trends in sub-Saharan Africa from 2010 to 2017.

The goat population in the Eastern and Western regions of the SSA have shown a continuously increasing trend from 2010 to 2017 (Figure 1). In Central Africa, goat populations marginally increased from 2010 to 2014 after which a drastic increase was observed from 2015 to 2017. The Southern Africa goat population is on the decline at an annual rate of about 1.77%. Like the trends of the general goat population, the trend of the population of goats for milk is increasing in Western and Eastern Africa. Central and Southern Africa regions have had a fairly constant goat population over the years (Figure 1). Of all the regions in SSA, Eastern Africa reported the highest increase in the population of goats used for milk production. This could be attributed to the aggressive promotions by the non-governmental organizations and bilateral agencies on adoption of dairy goats [7,11,12].

Total annual goat milk production and daily production per doe in SSA is presented in Table 2. Milk production was highest in Eastern Africa and lowest in Southern Africa. Eastern and Central Africa experienced an increase in milk production from 2010 to 2017 at a rate of 77,515 tons and 25,489 tons annually, respectively. Total annual milk production in Western and Southern Africa regions declined with time at a rate of 21,076 tons and 820.49 tons respectively.

Dairy goat productivity remained fairly constant in the Eastern region throughout the review period. This implies that the observed increase in annual production is as a result of the increase in the number of goats in the region (Figure 1). Owing to the increase in competition for production resources, e.g., land, this trend is not sustainable. Consequently, there is need to enhance productivity of the animals by improving management as well as through selective breeding. Dairy productivity in Western and Central regions declined while increasing trends were observed in Southern Africa. Dairy goats in Southern Africa show superior performance to goats in other regions in SSA.

### Dairy goat management systems

Effective management of dairy goats requires a holistic approach where issues pertaining to the production system, feeding and nutrition, disease management and breeding are adequately addressed. Dairy goats are mainly raised under smallholder mixed crop-livestock systems [3,9]. These systems vary depending on input use ranging from the low input systems to high input systems along a continuum. The level of input use influences the general management of dairy goats between systems. Shrestha and Fahmy [13] observed varied management practices, production environments and socio-cultural attributes of goat keeping households which demonstrate the complexity of goat production systems. The low input systems constitute the largest proportion of dairy goat production systems [9,14,15]. These systems are characterized by improvised housing and feeding infrastructure [2,16]. Most of the farms use raised slatted floor housing with wooden walls although in some cases earthen floor and concrete floor houses are used [17]. On the other hand, a few large-scale commercial goat farms exist mainly in Southern Africa and peri-urban areas of Kenya. These have a niche market for goat products in the urban centers [12].

Feeding of dairy goats is mainly forage-based where use of homemade rations dominates. In the majority of systems, the goats are fed on fresh grass fodder, forage legumes and pasture (Table 3) in stalls [16,20]. The availability of the fresh fodder and pasture varies seasonally depending on rainfall patterns. During dry seasons, the major feed resources used are crop residues and hay. The crop residues used vary from one region to another depending on the crops grown (Table 3). However, millet and wheat residues were common across in SSA. These residues are normally treated with molasses and urea prior to feeding. It is reported that, dairy goats are grazed in pastures together with sheep and/or other large stock or sometimes tethered in some systems [14,19]. Table 3 presents feed resources used in dairy goat diets in Eastern, Western and Southern sub-Saharan regions.

Supplementary feeding of dairy goats is a common practice among goat keepers. The main source of supplementation is a variety of agro-industrial by- products (Table 3) such as maize and wheat brans, cotton seed, groundnut and sunflower seed cakes etc. which are given to lactating goats. There are a variety of forage legumes and grasses used as supplementary fodder for goats. Minerals and vitamins are supplemented through vitamin premixes and mineral blocks mainly to the exotic dairy breeds. Mineral and vitamin supplementation is largely absent in systems where indigenous goats dominate.

Goats raised for milk production in SSA are comprised of indigenous and exotic breeds, and their crossbred genotypes [3,8,27]. The indigenous goats are predominant in the dry rangelands (arid and semi-arid regions) ofSSA, and are used for milk production besides meat and skin by the pastoral producers [3]. Some of the breeds found within these regions include the West African Dwarf, Sokoto Red and West Africa long-legged goat or the Sahel goat in west Africa; the multipurpose indigenous veld goats of South Africa and the pastoralist milking goats of the Gabra, Samburu, Orma, Somali and Rendille communities in Eastern Africa [3,8,28,29]. These goats are not subjects of selection since producers who keep them are keen on maintaining the adaptability of these breeds. Consequently, their milk production performance remains low. Improvement of these breeds for productive traits could however be achieved through within breed selection in their production environment.

In sub humid and humid regions of SSA, exotic breeds and their crossbred genotypes dominate. These breeds are highly productive but ill adapted to tropical conditions. They include Saanen, Alpine, Toggenburg, and Anglo-Nubian. The breeds have been the force behind the spread of dairy goat production in the SSA [8]. They were introduced through a variety of development interventions aimed at poverty alleviation and enhancing food security [30]. In the majority of these programs, crossbreeding to upgrade the indigenous breeds was preferred because of the fast results, although in some programs, purebreeding was used. Though the programs had implementation plans, there were no well planned breeding programs to sustain the achievements beyond the project periods. In some programs however, producers that were project beneficiaries evolved into organized breeding groups and breeder associations/societies e.g. the Dairy Goat Association of Kenya and the Saanen Goat Breed Society in South Africa, among others [9,31]. As a result, only in a few cases where crossbreeding was involved can the level of crossbreeding and the constitution of the genotypes be ascertained. Overall, there is minimal improvement that can be ascribed to selective breeding within the dairy goat systems in SSA. This minimal improvement has stifled dairy goat production and products diversification. For instance, when the available quantities of milk is low the propensity to produce varied products (such as yoghurt, skim milk, whole milk, low fat milk, various types of butter and cheese) from this milk is also low as well.

The primary products from a dairy goat system are milk and meat. Goat milk is marketed mainly through informal channels by selling directly to consumers at farm gate or at fresh markets [31]. However, processed goat milk and cheese is slowly making entry into the local supermarkets [7,31]. There is therefore the need for further sensitization on the nutritional and medicinal advantages of dairy goat products reported by Anaeto et al [28] and Mestawet et al [29]. The dairy goat product market is fairly developed in developed countries. Niche markets with clearly defined channels have been developed for dairy goat products and there are many reports of increase in the demands for the products [32].

## MAJOR CONSTRAINTS TO DAIRY GOAT PRODUCTION

To enhance the development of the dairy goat subsector, it is imperative that concerted efforts are made to alleviate the constraints that stifle its growth. Constraints to improvement of the dairy goat production can be categorized into nutrition and feeding, breeding and reproduction, diseases, parasites, climate change, and underdeveloped dairy goat products market [7,20,31,33,34].

Feeding is a key component of livestock management that influences animal performance. Livestock productivity can be increased three-fold by improving feeding to meet the nutritional requirements of the animals. In the dairy cattle sector the nutritional and feeding requirements of dairy cows are known to the level of stage of lactation for which concentrate feeds are available. Nutritional requirements of dairy goats are only available for the various classes of goats such as kids and mature does. Requirements for breeds, levels of productivity and environmental conditions of SSA have not been well studied. As a result, there are no standard concentrate containing rations for dairy goat available in SSA. Supplementary feeding is therefore based on agro-industrial by- products, leguminous trees/shrubs and grass fodder [16,19,20,26]. The quality of the agro- by products is highly varied depending on the type of the agricultural products and the processing methods used. Equally, the quality of the fodder and their availability is highly dependent on climatic and edaphic factors ultimately affecting dairy goat performance [11,20,35].

Though performance in animals could be enhanced through improvement of the production environment, this improvement is temporary. Selective breeding aims to confer permanent and heritable changes in the population. Goat breeding happens to a limited extent in SSA. Availability of breeding stock, inadequate recording infrastructure and absence of a breeding program with clearly defined objectives are among the major constraints to goat improvement through selective breeding [7,11]. A breeding program is necessary for ensuring that a program does not regress on its overall objectives. It is critical in determining whether the program seeks to have only a pure breed, crossbreed or synthetic breed, and for which environment as well as which traits to improve [27]. Breeding structures necessary for genetic improvement of goat genetics are scarce and where available, the prices are fairly exorbitant. Use of reproductive technology to disseminate superior genetics in SSA remains limited either due to unavailability of the necessary infrastructure or requisite manpower. As a result genetic improvement of dairy goats is still based on the conventional methods which undermine improvement of dairy goat performance through selective breeding [8].

Climate change impacts influence dairy goat performance just like other livestock species [34]. Rainfall intensity, variability and availability as well as temperature parameters affect availability of feed, disease and parasite epidemiology and response of animals to animal health management measures. Goat production systems in SSA are climate dependent, as a result, perturbations of the weather result in variation in productive, reproductive and adaptive performance of the goats. Consequently, this affects the quality and quantity of products from the dairy goat enterprise.

Milk is a perishable product. Therefore, organized and efficient collection, cooling and marketing systems are crucial to the overall viability and profitability of commercial dairying [36]. These are however lacking in most parts of SSA. Dairy goat products are sold primarily through informal marketing channels as fresh milk owing to the unstructured nature of the markets of these products [29,31]. These markets are highly inefficient due to improper market information and volatile product prices. While clear policy guidelines and structures exist on marketing of cow milk, these are lacking in most countries in SSA for goat milk. As a result, standards to ensure product quality from dairy goat systems are non-existent. Besides, there are no large scale goat milk plants; hence, value addition is largely done using traditional methods in small cottage industries set up within given locale [6,29]. Other constraints to dairy goat development include inability of goat farmers to produce milk consistently with local breeds and with good sanitary quality, low acceptability of goat milk in various cultural and taste habits when cow milk is cheaper to produce and consume, and lastly the unfavorable price relationship between goat milk and cow milk [6].

## PROSPECTS FOR RESEARCH AND DEVELOPMENT

Research forms the basic tool for improvement of any given system. It avails information and enhances knowledge in a particular area thereby offering solutions to problems in the system. The higher the rate of generation of knowledge in the system, the higher is the rate of development. Many times, scientific technologies do not reach the intended users or if they reach them, the rate of adoption is very low. This may be due to lack of cooperation between the scientists and other stakeholders in the value chain including farmers. Therefore, the involvement of policy makers, extension workers and planners is critical. Sustainable research and development programs for dairy goat production are those that are based on the existing practices and resources aimed at meeting the priority needs of the target groups. This calls for collection of baseline socio-economic data.

In every system of production, failures of intervention measures stick in the memories of the producers. Memories of previous failures inhibit acceptance of any new idea. As a result, evaluation of intervention measures needs to be done to ensure that the rate of success guaranteed is higher than failure. Therefore, it is important to encourage progressive development by providing solutions to the important impediments to dairy goat production through participatory research and development.

In order to achieve rapid development in the dairy goat subsector, research and development initiatives should be directed towards alleviating the aforementioned hurdles in nutrition and feeding, breeding, disease management and environmental resilience as well as dairy goat product markets. Feeding constitutes a substantial proportion of the cost components of any livestock production system. Feed constraints are in terms of both quality and quantity. Attention should therefore be given to ensuring a sufficient supply of good quality feed resources. Dairy goats are fed on fresh grass fodder, forage legumes and pastures in stalls. The availability of these feeds is seasonal and during the dry seasons, crop residues and hay that are of low nutritional value are available for use. Availability of goat feed could be enhanced through use of technologies such as hydroponics and irrigation of pastures and fodders. Efficiency in the utilisation of the high roughage tropical pastures can be achieved through use of better harvesting and preservation techniques and adequate understanding of the rumen ecology so as to easily manipulate the rumen environment for the benefit of the animal. Alternatively, attempts should be made at breeding of animals that can efficiently utilize high roughage tropical pasture. There is need for more investment in scientific research in nutrition and feeding to establish the nutritional requirements of dairy goats at various physiological growth stages in the tropics. Availing these requirements will set the stage for the feed processor to formulate rations and produce feeds that meet the performance requirements of the goats. Emphasis should be on utilisation of alternative sources of nutrients e.g. leguminous trees/shrubs as sources of protein.

Availability of breeding stock in SSA remains a limiting factor to breed improvement in dairy goats. This is primarily because there is still over- reliance on natural mating and use of fresh semen for artificial insemination. There is therefore the need to develop and avail extenders for goat semen that will sustain the vitality of the spermatozoa even after laboratory processing and preservation. The observed productivity differences between the regions (Table 2) presents an opportunity where Southern Africa could be a cheaper source of breeding stock to other regions. This is however limited by availability of suitable technologies of germplasm preservation.

Organized breeding programs need to be put in place with participation of the key stakeholders [7] to establish sustainable breeding schemes that will ensure dairy goat improvement through selective breeding. These programs should endeavor to use high throughput methods of genetic improvement such as genomic selection targeting productive and functional traits so that breed adaptability to production conditions is assured. This should be done bearing in mind both crossbreeding and purebreeding strategies to achieve short-term and long term gains of the program. This could be achieved through organizing producers into groups of breeders and multipliers [37].

The effectiveness of genetic improvement programs lies in the accuracy of selection of the animals, which is guided by precise estimates of breeding values. Estimation of breeding values requires adequate records. Such records are scarce because of the resources required for their collection. This, therefore, calls for urgent development, evaluation and application of simple performance and genetic evaluation procedures that take into account the needs and aspiration of goat keepers. These procedures should be affordable and simple enough to be applicable by a broad range of dairy goat producers. For instance, mobile phones are very popular with farmers in SSA, therefore development and deployment of mobile phone data capturing applications could go a long way in alleviating data recording problems among goat farmers. In addition, there is need to put in place effective feedback mechanisms to sustain the recording program.

High prevalence of diseases and parasites are a serious constraint to goat production, particularly in the more humid areas. These cause high mortality amongst kids, diminishing benefits of the goats’ high reproductive performance. In addition, external parasites weaken animals and thus cause extensive production losses. Ticks and mites are the most serious external parasites and transmit endemic Haemoparasites. Internal parasitism also ranks high among the factors that limit the productivity of goats although its effect is often underestimated. Other diseases that limit the productivity of goats in SSA include, among others, pneumonia, coccidiosis, contagious caprine pleuropneumonia, ecthyma, caseous lymphadenitis and brucellosis. The prevalence of these diseases has been difficult to quantify due to the lack of precise statistical records. Therefore, it is important to develop and validate disease diagnosis, monitoring and control methods specific to dairy goat production in order to reduce the mortality rates. There is need to carry out research on the methods of disease control, diagnosis and epidemiology of some of the diseases. In some communities in the SSA, there is use of traditional herbs in the treatment of some diseases. These herbs need to be investigated to determine their effectiveness and how they could be utilised better. Enhancement of farmers’ knowledge in aspects of disease control and management through community-based animal health organisations, farmers’ field days and ensuring that only qualified veterinary personnel are allowed to offer veterinary and pharmaceutical services could be tangible alternatives.

Goats are considered to be resilient when they are able to subdue strenuous conditions (perturbations) to survive and remain productive [38]. When the resilience of the goat traverses production environments, then it is said to be robust. Intrinsic robustness refers to the animal’s ability to invoke their natural biological and physiological processes to remain functionally productive under a variety of production conditions [39]. For instance, a goat is said to be robust when it produces during favourable conditions and sustains the production under strenuous circumstances. Strategies of goat breeding in low-input systems that emphasize on more robustness are a challenge now more than ever. This is because of the global environmental changes that have exacerbated environments heterogeneity, low feed availability and myriad stresses to the animals. These conditions can be managed to sustain production through efficient management systems as well as selective breeding for traits that enhance robustness such as feed utilisation efficiency. Adoption of efficient management systems are well beyond the economic ability of many goat producers and contravene some values of their production system. This leaves only selective breeding as the option to attain robustness in goat populations. It is however not within the realm of the systems values that all selective breeding approaches may be acceptable in the low input goat production systems.

A gap exists on dairy goat product development as evidenced by the low level of product diversification. There is therefore the need to conduct studies on dairy goat product, markets, and consumer preferences to provide information for the development of the marketing channels for these products. Small-scale producers should be encouraged to form groups to enhance their market bargaining power, access credit by provision of group collateral and interaction between the farmers thus enhancing information transfer. This will improve the production levels due to the credit incentives. Policies governing trade and marketing should be reviewed to protect the producer from extortion and exploitation by middlemen and enhance market competition.

There is need for the government, non-governmental organisations, international agencies and donors to provide interested people with institutional support by providing easy access to information relating to dairy goat production. This calls for sufficient stakeholder training in matters related to dairy goat production. When deciding on which training to offer, it is therefore important to consider the mode of communication and the gender targeted besides the traditional customs. Training packages should be comprised of information about disease management, feeding, breeding management and marketing.

## CONCLUSION

Dairy goats have gained importance in smallholder farming systems and in high potential areas due to increasing human population density and subsequent reduction in land size as a result of fragmentation. Generally, they contribute greatly to the meat, milk, fibre, pelts and skins used in SSA. The relative importance of each of these products varies from one region to another due to ecological, economic and cultural factors. However, the dairy goat sector has continued to perform below its potential due to the associated constraints mentioned above. It is important that the production environment is improved so that there are proper feeding and good husbandry practices that will enable the dairy goat to exploit its full genetic potential. Strategies of dairy goat breeding in low-input systems that place more emphasis on robustness are a challenge now more than ever, owing to global environmental changes. Therefore, to improve robustness, breeding should aim at increasing versatility of goats to the changing environments. To improve dairy goat production, there is need for a well-developed dairy goat product markets with more specialisation into market-oriented objectives where there is high contact between the production system and market. Consumer demands will consequently have a large impact on the systems. Therefore, to improve the current status of the dairy goat sector in SSA, individual and national efforts are required from all the stakeholders if any feasible self-sufficiency in dairy goat products is to be achieved in the foreseeable future.

## Figures and Tables

**Table 1:** Population of goats and their proportionate distribution in sub-Saharan Africa

SSA region	Total goat population	% of total goat population in SSA	Population of goats for milk	% Milk goats to the regional total
Eastern Africa	141,945,838	38.08	25,279,669	17.81
Western Africa	165,196,182	44.32	29,318,272	17.75
Central Africa	55,529,942	14.9	4,858,491	8.75
Southern Africa	10,044,078	2.69	110,693	1.10
Total	372,716,040	100	59,567,125	15.98

SSA, sub-Saharan Africa.

FAOSTAT [10].

**Table 2:** Doe milk production and productivity per year in SSA between 2010 and 2017

Year	Eastern		Western		Central		Southern	
			
	Production (tonnes)	Productivity (kg/animal)	Production (tonnes)	Productivity (kg/animal)	Production (tonnes)	Productivity (kg/animal)	Production (tonnes)	Productivity (kg/animal)
2010	1,132,659	67.2	1,218,992	58.2	120,808	64.8	15,631	114.8
2011	1,141,483	64.2	1,272,879	58.3	123,667	64.9	17,947	110.6
2012	1,608,553	66.9	1,300,992	57.4	125,537	65.6	18,107	110.4
2013	1,624,316	65.9	961,510	40.8	124,549	65.0	18,197	110.3
2014	1,678,020	65.4	1,039,225	41.1	125,882	65.1	13,930	118.4
2015	1,647,316	65.6	1,110,805	41.8	252,511	57.1	11,666	124.2
2016	1,672,689	65.6	1,129,957	41.0	263,577	56.4	12,168	122.8
2017	1,659,116	65.6	1,138,576	38.8	272,137	56.0	13,283	120.0

SSA, sub-Saharan Africa.

FAOSTAT [10].

**Table 3:** Feed resources used in feeding dairy goats in sub Saharan Africa

Region	Feed resources				Source

	Fodder	Agro industrial by-products	Pastures	Crop residues	
Eastern Africa	*Melia azedarach* leaf meal, *Ficus* spp., Leucaena, Calliandra, Mulberry, Grevellia, Gliricidia, Sesbania, Tithonia, *Lantana camara*, Siratro, Sweet potato vine, *Clitoria tarnatae*, Lucerne, Desmodium, Napier grass, Sorghum	Sunflower seed cakes, Groundnut cake, cotton seed cake, Sunflower cake, Maize germ and bran	Rhodes grass, *Panicum* spp., *Cenchrus* spp., Bana grass	Maize and Sorghum stover, millet straw, Rice straw, Wheat straw, Barley straw, Oat straw, bean haulms, Sugar cane tops, Sunflower heads	Nampanzira et al [16], Kaberia et al [17], Bett et al [9], nafis.go.ke/livestock/dairy-goat-production/feeding/ [18]
Western africa	*Gliricidia sepium, Leucaena leucocephala, Centrocema pubensce, Moringa oleifera, Afzelia Africana, Ficus gnaphalocarpa, Annona senegalensis, Arachis hypogaea, Pericopsis laxiflora, Pterocarpus erinaceus, Acacia albida*, (pods)	Brewers’ dried grains, Wheat bran, Pearl millet bran, Sorghum bran,	Natural pastures, Natural pasture-hay, *Andropogon gayanus, Eragrostic tremula, Pennisetum pedicelatum* and *Digitaria ciliaris*	Groundnut haulms, Cowpea husk, Urea treated Millet straw, Sorghum stover, sesame, cassava peels, wheat straw and Okra leaves	Partey et al [19], Amole and Ayantunde [20], Babale et al [21], Ikyume et al [22] Agossou et al [3], Duku et al [23]
Southern Africa	*Cajanus cajan, Cassia rotundifolia, Desmodium* spp., *Leucaena* spp., *Lotononis bainesii, Macroptilium etropurpureum, Macrotyloma azillare, Neonotonia wightii, Stylosanthes* spp., *Trifolium semipilosum, Vachellia karroo* leaf meal	Rice bran, Millet bran, Brewer’s grains, Orange wastes, Lemon wastes, Pineapple waste, Molasses, Cotton seed cake, Wheat bran, Barley bran	*Themeda triandra, Panicum* spp., *Hyparrhenia* spp., *Andropogon* spp., and *Heteropogon contortus*	Maize stover, Rice straw, Millet straw, Wheat straw, Barley stover, Groundnut hulls, Sunflower hulls, husks and cake, Sugercane tops, Oats stover	Kadzere [24], Chakeredza et al [25], Idamokoro et al [26]
